# Synthesizing Genotoxicity Results in the MultiFlow Assay With Point‐of‐Departure Analysis and ToxPi Visualization Techniques

**DOI:** 10.1002/em.70003

**Published:** 2025-03-13

**Authors:** Yusuf Hussien, Stephen D. Dertinger, George E. Johnson

**Affiliations:** ^1^ Instiutue of Life Sciences Swansea University Swansea UK; ^2^ Litron Laboratories Rochester New York USA

**Keywords:** benchmark dose analysis, data visualization, flow cytometry, genotoxicity, hierarchical clustering, ToxPi

## Abstract

In vitro genotoxicity has historically served a hazard identification role, with simple binary outcomes provided for each of several single endpoint assays. This will need to change, given: (i) efforts to curtail animal testing, (ii) the increased use of multiplexed in vitro assays and the ongoing development of NAMS, and (iii) the desire to holistically consider quantitative results from multiple biomarkers/endpoints that take potency into consideration. To help facilitate more quantitative analyses of multiple biomarkers and/or assay streams, we explored the combined use of PROAST and Toxicological Prioritization Index (ToxPi) software. As a proofofconcept, this investigation employed the MultiFlow DNA damage assay, focusing on γH2AX and p53 biomarkers at two time points, whereby 10 genotoxicants were evaluated in the presence and absence of rat liver S9 metabolic activation. Whereas PROAST was used to calculate BMD point estimates and confidence intervals (CIs), ToxPi synthesized the BMD results into visual, quantitative summaries conveying genotoxicity and metabolic properties. Our analyses suggest that ToxPi's data synthesis and visualization modules provide useful insights into compound response, chemical grouping, and genotoxic mechanisms. By integrating multiple data sources, we find that ToxPi offers a powerful complementary approach to traditional BMD CI graphs, particularly for the simultaneous analysis of multiple biomarkers, enhancing chemical potency analysis of complex datasets.

## 1 Introduction

A variety of exogenous and endogenous sources of DNA damage, such as ionizing radiation, reactive oxygen species, replication errors, alkylating agents, and carcinogenic compounds, introduce mutations that lead to defective genetic information, faulty cell development, impaired protein production, damaged cellular function, cellular apoptosis, genetic conditions, and tumorigenesis, ultimately affecting the survival of humans and other organisms (Wilson and Hunt 2002; Song et al. 2015; Chatterjee and Walker 2017). Base excision repair, nucleotide excision repair, and mismatch repair are all mechanisms activated once DNA damage is detected at genetic checkpoints and help to cleave/excise mutations from genetic material (Hakem 2008). Using diverse assays and testing models to detect DNA damage and repair events is crucial for designing effective protocols to identify potential sources of genotoxicity (Kim et al. 2013).

Genotoxicity tests have been used for decades as chemical hazard identification tools, meaning they were used to provide a binary outcome on compound genotoxicity—yes or no. However, there is growing recognition among pharmacologists, toxicologists, and pharmaceutical regulators that this approach overlooks the ability of genotoxicity tests to provide detailed dose–response information and insights into mechanisms of action, which can significantly enhance risk assessments. This shift in perspective has been driven by advancements in experimental designs and data acquisition methods, which now enable the production of reliable and quantifiable dose–response information. This makes genotoxicity test results a valuable addition to risk assessments by providing more nuanced and actionable insights surrounding potential chemical hazards and their underlying genotoxic mechanisms (MacGregor et al. 2015; Heflich et al. 2019). Such an expanded use of genotoxicity data, including in vitro data, seems important, given the many initiatives aimed at curtailing animal testing, and integrating quantitative genotoxicity data into risk assessments helps meet the broader scientific and ethical demand for more predictive, human‐relevant testing methods. This shift represents a step toward achieving more comprehensive safety evaluations while advancing the vision of an animal‐free future in toxicology.

An important requirement for using genetic toxicology results for more than hazard identification purposes is that there must be a way to reliably evaluate dose–response relationships. Modern dose–response analysis metrics, such as the No Observed Genotoxicity Effect Level (NOGEL), offer an approach that identifies the highest dose at which no genotoxic effects are observed in a given experimental setup (FDA 2002). While useful in determining safety exposure levels, it only considers the doses tested in the experiment and does not account for the full dose–response relationship in a continuous manner.

The benchmark dose (BMD) model is a modeling approach that describes the dose (or concentration) that produces a predetermined change in response to a chemical being studied (also known as the critical effect size, or CES) to generate a statistically robust point‐of‐departure (PoD) metrics. Particularly, it produces upper and lower confidence interval bounds—the BMDU and BMDL, respectively—providing insight into model uncertainty and variability (Davis et al. 2011; Sturla 2018; FDA 2002; Dorato and Engelhardt 2005). The BMD also leverages the entire dose–response dataset to offer greater precision and flexibility regarding experimental design and dose selection, making it a more reliable tool in hazard characterization and information extraction from highly detailed dose–response datasets (Davis et al. 2011).

One of the tools used to extract the BMD from dose–response data is PROAST (v70.3): an R Gui–based (v4.2.2–4.4.0) software package that uses statistical modeling algorithms, such as the nested exponential/hill four‐parameter models, to mathematically analyze concentration–response datasets (Wills et al. 2017). Based on a user‐defined critical effect size (CES) that describes a predefined change in response above the mean background control, PROAST calculates BMD, BMDL, and BMDU metrics, and provides them in tabular form (RIVM 2021). The package also includes several useful visualizations that aid in the interpretation of datasets, providing quantitative data on compound potency and toxicity.

However, one complication to the use of PROAST and other BMD software packages, and for making effective use of quantitative genotoxicity data in general, is the increasing use of multiplexed in vitro assays, and/or the desire to integrate multiple data streams into an assessment. BMD software does not ordinarily accommodate multiple endpoints/biomarkers; rather, it focuses on a single response at a time. While the so‐called covariate analysis option allows a user to simultaneously fit multiple curves, an underlying requirement is that the response metric is the very same, single endpoint/biomarker. This limitation highlights the need for complementary approaches capable of integrating and visualizing data from multiple biomarkers to provide a more holistic assessment, such as the Toxicological Prioritization Index (ToxPi) software.

ToxPi (Version 2.3: Marvel et al. 2018) is a JavaScript visualization tool designed to translate quantitative toxicity data into a single ToxPi score, ranking compounds on a continuous spectrum to better discriminate between potency groups (Reif et al. 2012). One of its greatest strengths is its ability to simplify and effectively integrate results from diverse assays or multiple biomarkers within a multiplexed assay. The tool generates ToxPi profiles—dynamically created pie charts that visually represent the distinct components and features of each dataset. Additionally, ToxPi incorporates hierarchical and K‐means clustering algorithms using agglomerative nesting (AGNES) and principal component analysis (PCA) algorithms to group compounds based on similarities across the biomarkers assessed (Marvel et al. 2018).

The work described herein makes use of previously published MultiFlow assay data (Tian et al. 2020) and focuses on γH2AX and p53 biomarkers at two time points across 10 known genotoxicants with varying modes of action. Advantages realized through the combined use of PROAST and ToxPi are discussed to evaluate whether both applications can comprehensively compare and visualize original dose–response metrics for both parent compounds and their metabolites using a unique protocol described here.

## 2 Methods

### 2.1 In Vitro MultiFlow Datasets

The in vitro MultiFlow assay experiments were performed by Litron laboratories from their previously published article by (Tian et al. 2020) where 15 compounds were analyzed using their MultiFlow DNA damage kit—measuring the activation of p53 as a nuclei‐free measurement, phosphorylation of H2AX at serine 139 (γH2aX) to detect double‐stranded DNA breaks, and phosphorylation of H3 at serine 10 (p‐H3) as a mitotic cell activity endpoint at 4/24‐h timepoints, in the presence and absence of low dose 0.25% vol/vol S9 rat liver to measure metabolic activation effects. Methodologies and modeling used are further detailed in previous publications performed by (Slob and Setzer 2014; Bryce et al. 2016, 2017). As described in (Tian et al. 2020), a CES of 0.3 was chosen, representing a 30% change in response relative to the control, with exception to the RNC endpoint which had a value of −0.3. A summary of the 10/15 genotoxicants from the (Tian et al. 2020) study assessed in this analysis is listed in the Table 1, providing further insight into their respective modes of action and metabolic activation properties (including sources/citations).

**TABLE 1:** Test compounds, sources, modes of action, and metabolic properties.

Chemical (abbreviation)	CAS number, source—Sigma Aldrich	Primary mode of action, metabolic properties and references
Mitomycin C	50‐07‐7	Clastogen/mutagen, DNA cross‐linking (dsDNA breaks), alkylating properties and oxidative damage stress (Kirkland et al. 2016; Higa et al. 1998).
Dibenzol[a,l]pyrene	191‐30‐0	Mutagen, polycyclic aromatic hydrocarbon, requires metabolic activation (thought to be primarily activated by CYP1A1, forming DNA adducts leading to mutations) (Arif and Gupta 1997).
Resorcinol	108‐46‐3	Clastogen (in vitro), in vitro mammalian cell positive (MLA assay with and without metabolic activation positive/in vitro human lymphocyte MN positive in absence of metabolic activation); in vitro findings not confirmed in vivo (Negative in mouse MN) (European Food Safety Authority 2010).
2‐Aminoathracene	613‐13‐8	Mutagen with some clastogenic properties, aromatic amine, requires metabolic activation (CYP1B1, 2A family) (Carriére et al. 1992; Helma et al. 2001).
Benzo[a]pyrene	50‐32‐8	Clastogen/mutagen, polycyclic aromatic hydrocarbon, requires metabolic activation and forms bulky adducts (CYP1B1, 2A family) (Ramesh et al. 2007; Kirkland et al. 2016).
7,12‐Dimethylbenzanthracene (DMBA)	57‐97‐6	Clastogen/Mutagen, requires metabolic activation (CYP1B1), forms bulky adducts (Kuklenyik et al. 2004; Kirkland et al. 2016).
2‐amino‐1‐methyl‐6‐phenylimidazo [4,5‐b] pyridine (PhIP)	105,650‐23‐5	Clastogen/mutagen, heterocyclic amine, requires metabolic activation (Kirkland et al. 2016; Krais et al. 2016).
Cyclophosphamide Monohydrate	6055‐19‐2	Mutagen/Clastogen, nitrogen mustard, requires metabolic activation (CYP2B6, CYP2C19, CYP2C9, and CYP3A4/5) (Kirkland et al. 2016; Rodriguez‐Antona and Ingelman‐Sundberg 2006; Khalil et al. 2018)
2‐Actylaminofluorene	53‐96‐3	Mutagen with some clastogenic properties, requires metabolic activation (CYP1A2), forms C8 adduct on guanine (Otteneder and Lutz 1999; Kirkland et al. 2016; Sugimura et al. 2011).
Diethylnitrosamine	55‐18‐5	Mutagen with clastogenic properties, nitrosamine, *O* ^6^‐alkylguanine DNA lesions. Requires metabolic activation (Bartsch and Montesano 1984; Yamazaki et al. 1992).

*Note*: Any abbreviations for compounds are included in brackets and will be subsequently used in the results/discussion sections.

### 2.2 PROAST Parameters—BMD Analysis

The (Slob 2002) paper states that for toxicological continuous endpoints, a single dose–response model is unable to fully encapsulate the underlying biological mechanisms, so the implementation of nested models is applied to the original concentration and induction values to map out the dose–response curve, thereby effectively estimating the BMDL and BMDU using the CES. Hence, BMD analysis was performed following the (Tian et al. 2020) protocol using PROAST v67.0 with conditions being outlined in the study and BMD figures from that study were collected for further visualization. The PROAST software package, employs nested Exponential and Hill coefficient models, known as the four‐parameter exponential models, to analyze concentration–response datasets for each chemical's biomarker to cultivate BMDL and BMDU (90% CI) values (using bootstrapping), providing a quantitative assessment of compound potency and toxicity (Wills et al. 2017; RIVM 2021). PROAST now supports model averaging, a modern alternative to the traditional Hill or exponential modeling approaches. This method reduces bias by accounting for variability across models, quantifies uncertainty through integrated predictions, and applies model weights based on fit, determined by Akaike or Bayesian Information Criteria. (RIVM 2021). These metrics can then be visualized to explain the dose–response curve of each tested compound, as endorsed by the European Food Safety Authority (RIVM 2021). A CES value of 0.3 was selected for both γH2aX and p53 biomarkers, while polyploidy was set at 5.0 with the S9 conditions being the covariate factor in the analysis, as described in (Tian et al. 2020). The dose and induction figures were selected as independent and response variables respectively and “exponential models 3 or 5 from various families of models” within PROAST was selected to fit the dose–response curve. For endpoints where the BMD was unable to be modeled (either due to response not reaching the CES or the true BMD estimate exceeding the tested concentration range), no figure was recorded. Where the BMDL and BMDU were calculated, the values were recorded for each chemical, biomarker, timepoint, and S9 activation properties.

### 2.3 ToxPi Visualization

BMDL and BMDU figures were further visualized using ToxPi: A JavaScript program that visualizes and interacts with quantitative mutagenicity data, translating it into a single ToxPi score that scales mutagenicity on a continuous spectrum instead of fixed groups to better differentiate toxicity levels (Reif et al. 2012). BMDL and BMDU data generated through PROAST are initially transformed by fitting the BMDs within a realistic concentration testing range of 0.0001–10,000 μM as recommended by guidelines for various in vitro genotoxicity assays (OECD 2016a; OECD 2016b; ICH S2(R1) 2012). Therefore, all values below 0.0001 μM and any figures exceeding 10,000 μM are subsequently transformed to 0.0001 and 10,000 μM, respectively. Both BMDL and BMDU metrics are incorporated into individual ToxPi endpoint slices, weighting folds percentages can be defined for each endpoint, and a −log_10_(x) transformation (to ensure more potent compounds (which would have lower BMD values) show as having higher ToxPi scoring) is applied, yielding ToxPi profiles (a profile that illustrates each compound and their potencies across all endpoints as a singular pie chart). The ToxPi score is a metric that is calculated when the sum of all input values across assays is transformed into a slice/component score for each compound. These scores are normalized between 0–1 by subtracting the minimum value and dividing by the range. The normalized scores are then multiplied by the user‐selected fold‐weight (or by 1 ÷ the number of slices if weights are equal), with the resulting component scores summed and ranged between 0–1, producing a final ToxPi score. This score indicates that compounds with values closer to 1 are more potent at inducing toxicity while those with values closer to 0 are less potent (Auerbach et al. 2016). These figures combine overall responses across all figures into an automatically generated visualized pie chart with 90% CI (through 1000 automated bootstrap runs) and features that allow for the toxicity figures for all compounds across all biomarkers/time points/metabolic conditions to be effectively compared, which are illustrated in the below.

## 3 Results

See Figures 1, 2, 3, 4, 5, 6, 7, 8.

**FIGURE 1:**
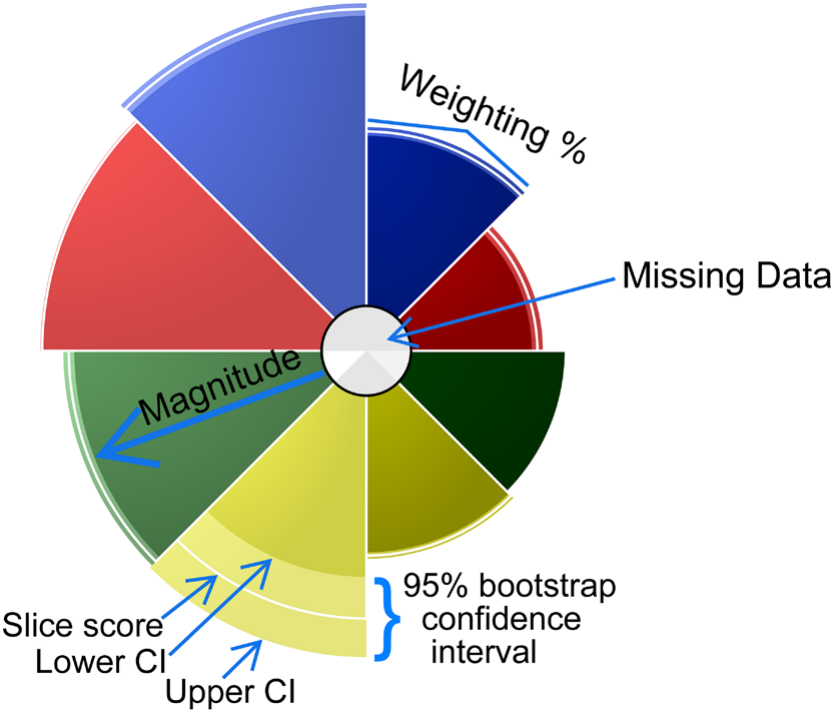
ToxPi Profile key illustrating the features and properties that produce the visual details for individual chemicals in dose–response datasets. The magnitude of each slice represents the relative risk once the −log_10_(x) transformation is applied, the width of each slice represents the weighting percentage applied to each slice, and the degree of graying within the center of the ToxPi profile represents the amount of missing data within the original dataset where increased darkening represents more missing individual data points. The 90% CI is represented by the pale section within the outer segment of each slice, where the inner edge represents the lower CI and is equivalent to the BMDU, the outer edge represents the outer CI and is equivalent to the BMDL, and the white line within the center represents the slice score for that endpoint. This profile was produced using MultiFlow data derived from the (Wheeldon et al. 2021; Tian et al. 2020) studies for the chemical resorcinol.

**FIGURE 2:**
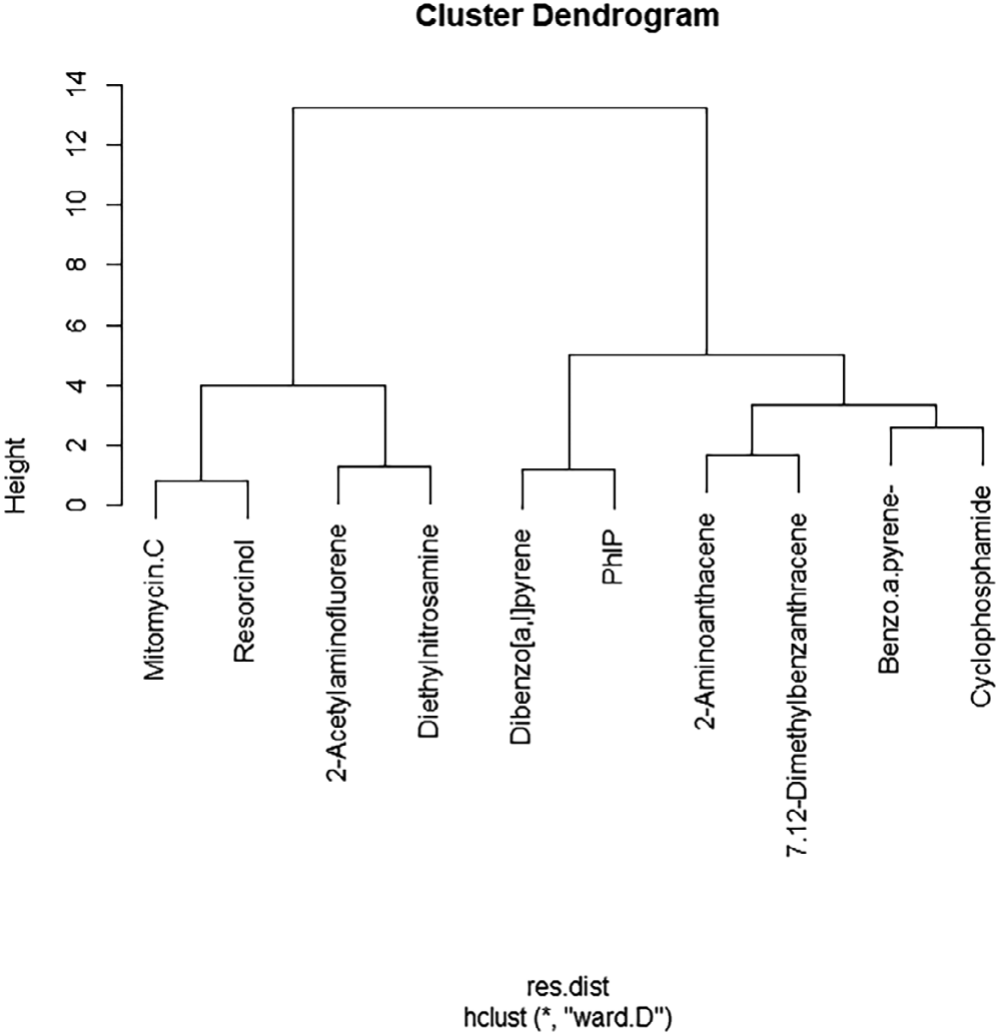
Euclidean Hierarchical clustering generated through R Gui using the *hclust* function ward. D clustering algorithm to show the distinctive groupings of each compound based on their S9 potency ratios CI range values, as described in (Wheeldon et al. 2021) using the divisive difference between the S9− and S9+ of each endpoint tested: 4 h γH2aX, 4 h p53, 24 h γH2aX, and 24 h p53. The branch height signifies the degree of relatedness among compounds, with shorter heights denoting a higher degree of similarity.

**FIGURE 3:**
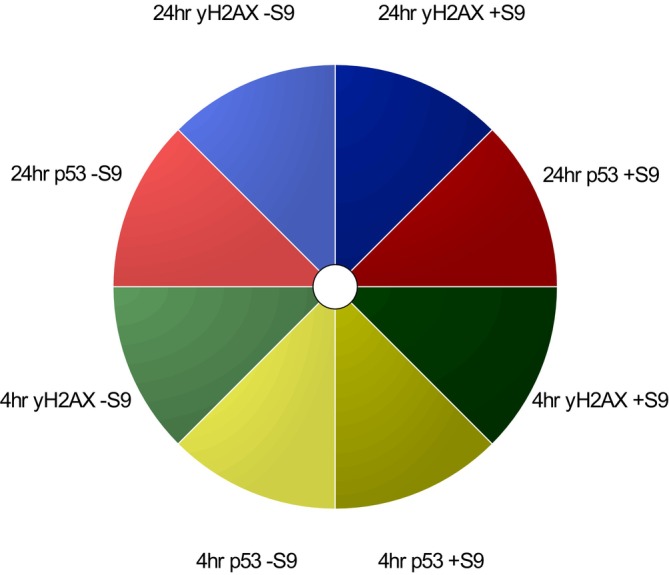
ToxPi profile key illustrating each slice with its respective endpoints, including color‐coordinated endpoints for γH2AX and p53 endpoints, at 4/24‐h timepoints with S9 present (S9+) and absent (S9−).

**FIGURE 4:**
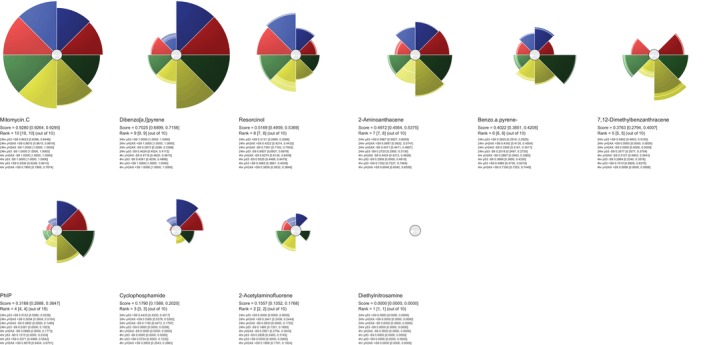
ToxPi profiles (with bootstrap CI) generated from BMD data for each chemical, showing the biomarkers and their ToxPi scoring (alongside bracketed CIs) for each compound across all endpoints. Each slice represents a biomarker at a given time in the presence/absence of S9. Shading in the center of the circle represents uncertainty for each slice (darker = more uncertain). The radius of each slice (from the center of each circle to either the edge or white line toward the edge of the slice) represents the response of each biomarker and the widths of the user‐defined weightings. The brighter regions presented in each are the bootstrap CI where the outer edge shows the upper CI and the inner edge is the lower CI for each slice.

**FIGURE 5:**
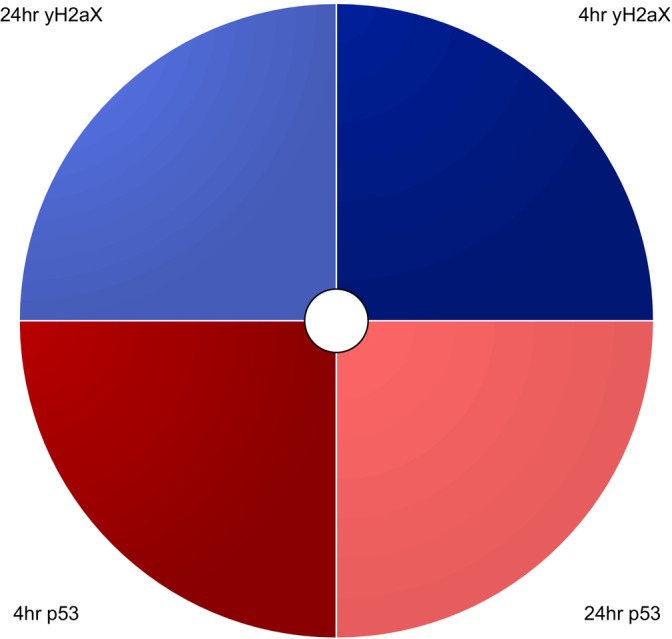
ToxPi profile key illustrating each slice with its respective endpoints, including color‐coordinated endpoints for γH2AX and p53 endpoints at 4/24‐h time points.

**FIGURE 6:**
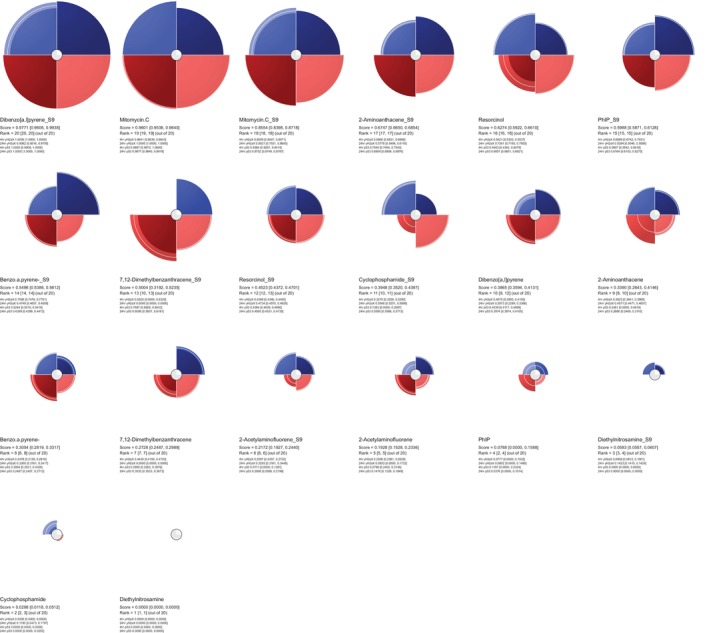
ToxPi profiles (with bootstrap CI) generated from BMD data for each chemical, showing the biomarkers and their ToxPi scoring (alongside bracketed CIs) for each compound across all endpoints. Shading in the center of the circle represents uncertainty for each slice (darker = more uncertain). The radius of each slice (from the center of each circle to the either the edge or white line toward the edge of the slice) represents the response of each biomarker and the widths the user‐defined weightings. The brighter regions presented in each is the bootstrap CI where the outer edge shows the upper CI, and the inner edge is the lower CI for each slice.

**FIGURE 7:**
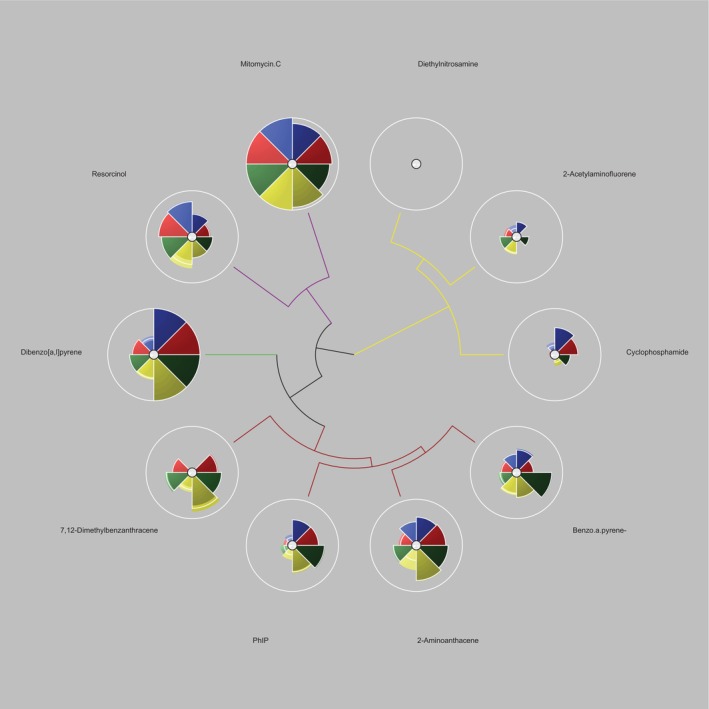
ToxPi profiles hierarchical clustering generated through complete divisive clustering to show grouping of each chemical, which are color‐coordinated. Green indicates compounds where genotoxicity was dramatically increased in the presence of S9 (average ratio of 2.6‐fold in ToxPi across all endpoints), red for compounds where S9 increased genotoxicity (average ratio of 1.6‐fold in ToxPi across all compounds/endpoints), yellow for no potency changes when S9 is introduced and purple, where S9 presence reduced genotoxic capacity of a compound; correlating with the S9 potency ratio CIs generated by (Wheeldon et al. 2021).

**FIGURE 8:**
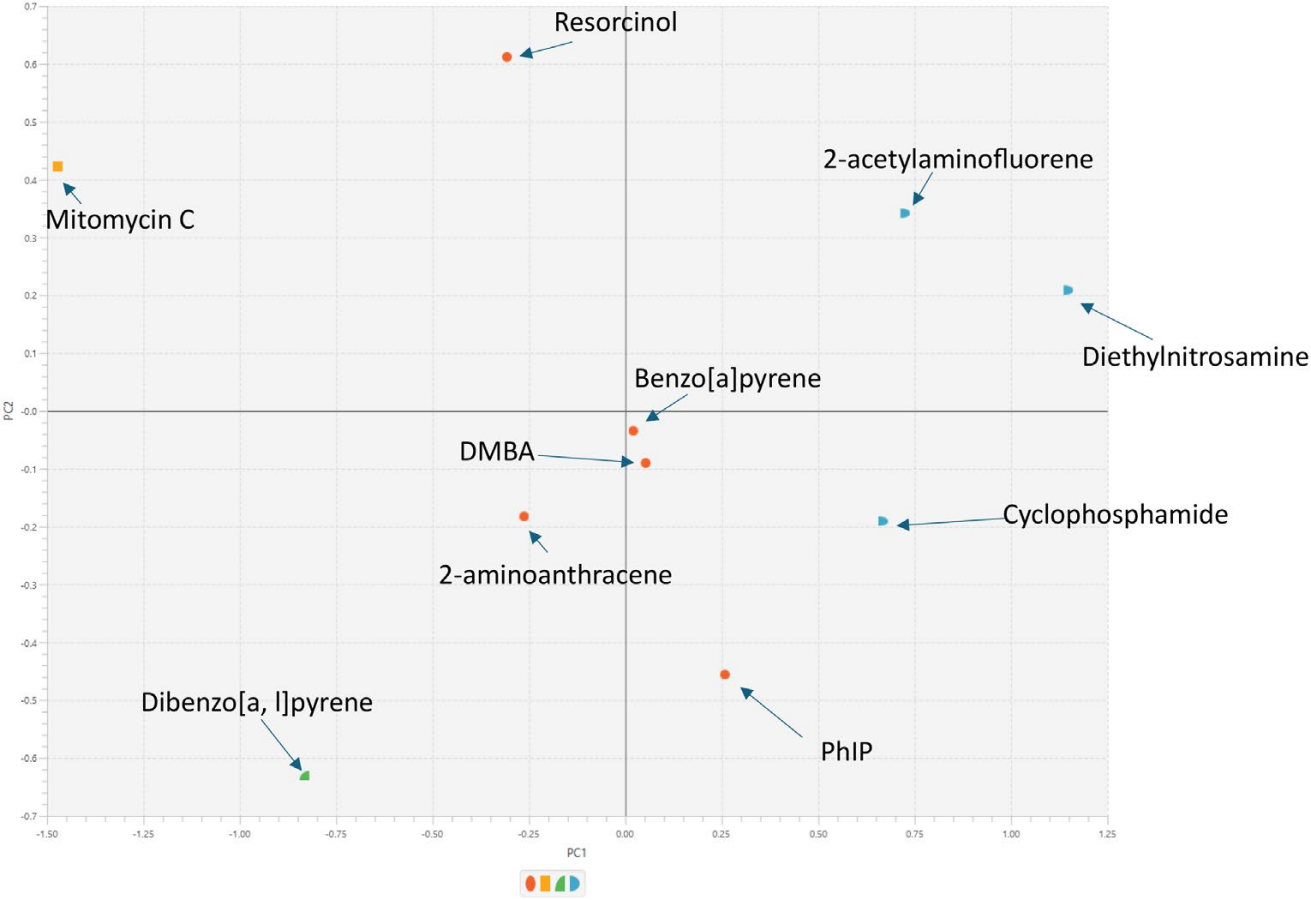
ToxPi K‐means AGNES/PCA clustering where each compound is grouped based on variation across the ToxPi scores/genotoxicity and biomarker sensitivity. Each point represents a compound that is automatically given unique colors/shapes based on a user‐selected number of clusters (4 clusters in this case) and presented in the graph legend. Each color/shape represents each unique cluster that compounds have been placed into. The pattern located is that PC1 compounds with higher ToxPi scores are toward the left, and for PC2, compounds with increased response in the presence of S9+ are at the bottom. Furthermore, the compounds cluster similarly in K‐means colors/shapes according to their metabolic properties as described in the hierarchical cluster chart, with the exception of mitomycin C and resorcinol: Both compounds that have reduced potency with S9 presence.

## 4 Discussion

Figure S1 presents BMD CI high‐low plots that illustrate significant differences in responses among chemical compounds by assessing the presence or absence of overlapping CIs between biomarkers. These figures were adapted from (Wheeldon et al. 2021) and utilized the BMDL and BMDU values to construct the high‐low plots. While the figure demonstrates some ability to rank potency and allows for comparison of compounds across different endpoints, its utility is significantly constrained by its limitation to a single endpoint at a time. This means that this approach fails to integrate all biomarkers, time points, and metabolic states into a single combined analysis figure used for comparative analysis, limiting the ability to directly compare overall compound toxicity and assess individual endpoint responses effectively. Instead, multiple endpoint figures must be assessed simultaneously, complicating efficient dose–response and MoA analyses for chemical or drug safety evaluations. Additionally, the use of BMD CIs for biomarker response assessment is debated as a single BMD figure is not determined. Instead, 90% CIs are generated, indicating the concentration range where the true BMD exists. This approach is reflective of the estimative nature of BMD modeling, providing a more robust measure of the PoD. It also fails to highlight how some endpoints are considered higher in significance across regulatory bodies and applies weighting applications to those biomarkers.

These restrictions highlighted here hinder the comparative capacity of multiplexed assays. However, combining BMD CI analysis with ToxPi visualization offers a more sophisticated, efficient, and comprehensive representation of responses across multiple biomarkers, effectively addressing the shortcomings of traditional BMD CI figures and modeling plots. BMD CI and ToxPi analysis allow for datasets modification, enabling the separation of S9+ and S9− figures for each compound. This depicts specific compound potencies in both metabolic states within a single figure. This is demonstrated in Figure 1, where differences in biomarker activation highlighted by the ToxPi analysis reveal response variations between compounds across both metabolic states.

ToxPi also enables users to customize the selection and weighting of individual slices, providing flexibility in generating ToxPi figures and tailoring weightings to specific biomarkers across multiple unique assays. As a result, biomarker responses and compound toxicity metrics can be more precisely aligned with the relevance of each endpoint in the context of each assay. This enhances analytical nuance by incorporating endpoint weightings that are tailored to regulatory frameworks. However, it is important to note that ToxPi does not automatically apply weighting according to regulatory knowledge. Instead, it serves as a platform for users to apply their knowledge and expertise in determining the relative importance of endpoints in different assays and to determine which weightings figures should be applied to each biomarker. This facet enables results that hold relevant weightings across endpoints to be more precisely determined.

Key findings from the clustering analysis include the strong association between Mitomycin C and resorcinol, both of which demonstrated reduced response in the presence of S9, thereby clustering closely together. Meanwhile, compounds such as 7,12‐dimethylbenz[a]anthracene (DMBA) and 2‐aminoanthracene, which exhibited increased potency in the presence of S9, also formed a distinct cluster. The S9+/S9− ToxPi profile results in Figure 4 reaffirm these findings as both mitomycin C and resorcinol have ToxPi scores higher in the absence of S9− compared to S9+. 2‐acetylaminofluorene and diethylnitrosamine, which showed no significant difference in biomarker activation with or without S9, clustered together as expected and ToxPi ranking also reflects this. Interestingly, both benzo[a]pyrene and cyclophosphamide showed similar, modest increases in activity with S9, consistent with ToxPi rankings and Euclidean hierarchical cluster figures. However, despite that ToxPi's hierarchical clustering algorithm (Figure 6) separated these compounds, their ToxPi profiles (Figure 2) suggest that they are more closely related than indicated by the Figure 5 clustering results, highlighting differences between ToxPi's divisive top‐down approach and the AGNES bottom‐up approach of the *hclust* function.

Similar to the compounds, PhIP and dibenzo[a,l]pyrene both show a significant increase in signal when metabolically activated, a trend reflected in both the hclust function and ToxPi ranking and clustering, despite what the ToxPi hierarchical clustering suggests, which left PhIP/dibenzo [a,l]pyrene and benzo[a]pyrene/cyclophosphamide in different grouped clusters. Additionally, it is worth noting that BMD CI limits were established in this analysis, and unlike the original study by (Wheeldon et al. 2021), a subtraction approach was utilized instead of division to assess S9 potency ratio CI differences. The absence of recorded RNC BMD CIs in this analysis, as opposed to the referenced study, likely further contributed to the variations observed in the clustering outcomes between the two studies. Further clustering analysis requires the integration of further programs to be performed effectively, and hence, direct raw figures can be compared using either R functions, JMP software's MDS platform, XLSTAT in excel, etc. The process further results in the transformation of the original data to fit the desired visualization and clustering purposes; hence, Gene ToxPi offers a robust alternative to these current approaches.

Despite a singular system being implemented to produce visualized ToxPi figures for the MultiFlow and MicroFlow assays, multiple assays are covered in the field of toxicology, pharmacy, pharmacology, and genotoxicology. This means unique approaches are required for the protocols described in this study when a different assay is considered for ToxPi visualization due to the different meanings behind the numerical values produced in raw figures, whether any significant differences are present in numerical units, the study design behind the analysis, and the formatting/transformations that are applied.

Diethylnitrosamine is a potent genotoxicant, yet its ToxPi score of 0.000 might initially appear to suggest a lack of response across all biomarkers. However, this outcome highlights the importance of marker selection and coverage when designing multiplexed assays, rather than the limitations surrounding the ToxPi framework itself. In this case, the lower ToxPi score stems from the absence of relevant gene mutation endpoints for diethylnitrosamine detection, resulting in elevated BMD values across endpoints. This necessitated the adjustment of some biomarkers to 10,000 μM, as detailed in the methods. The specific nature of O6‐alkylguanine DNA lesions, a hallmark of diethylnitrosamine's mutagenicity, likely necessitates specialized detection techniques, including probes, antibodies, or microbial assays, that fall outside the scope of the MultiFlow assay (Bartsch and Montesano 1984). While γ‐H2AX is capable of detecting DNA damage, it does not specifically target O6‐alkylguanine lesions, potentially contributing to the elevated BMD values observed. Consequently, the ToxPi score of 0.000 reflects diethylnitrosamine's comparatively lower potency within the chosen endpoints of this study, rather than a lack of genotoxicity. This suggests that there is a need for follow‐up testing (such as the Ames test) and careful endpoint selection to ensure adequate coverage, particularly when designing assays intended to predict responses across a broad spectrum of genotoxicants. However, it is important to note that the aim of this analysis was not to evaluate the predictivity of individual markers but rather to derive insights from BMD estimates across multiple endpoints. Both these additions to the existing methodology could provide additional key information to develop a more robust compound biomarker response profile.

Additionally, the ToxPi results generated are subjective to each dataset, and therefore currently, ToxPi scores themselves cannot be compared across multiple individual ToxPi analyses (you cannot currently compare one set of ToxPi scores to a separate set). However, as groupings are a collective result of all BMD input values and ToxPi profiles, approaches to combining the clustering results may be compared, and extensive results from a collective effort would alleviate these concerns. Furthermore, multiple analyses may be combined into a single assessment for extensive compound analysis, which has already been demonstrated in (Dertinger et al. 2024) which analyzed 126 compounds across 7 biomarkers. While the MultiFlow assessment process is straightforward, analyzing additional compounds and endpoints can complicate clustering by modes of action, reflecting study design challenges rather than limitations of ToxPi.

Weighting percentages are configured such that increasing the percentage enhances the priority or importance assigned to each endpoint/slice, directing greater focus toward higher‐weighted factors in the analysis. A standardized weighting percentage system has not been formally set; hence, it has remained unchanged across all endpoints as 1‐fold in this analysis (leaving all weightings as equal), as justifications for numerical selection, biomarker biological importance, and regulatory constraints are required before a standardized system of weighting fold applications may be set. Extensive experimentation is currently underway to refine model weightings and significance by evaluating assay‐specific differences in biomarker selection and response information. In the long term, this research seeks to establish a tailored, individualized approach that accounts for the unique contributions of each assay.

Although the data presented here focus on two endpoints (+/− S9), this analysis serves as an initial evaluation of the methodology, demonstrating its potential utility for more complex multiplexed datasets. The approach's adaptability to integrate multiple biomarkers and conditions makes it well‐suited for scaling up to multiplexed studies, and while traditional binary hazard assays might achieve clustering of compounds in the presence/absence of S9, such approaches lack the nuanced integration of biomarker‐specific responses across time points and metabolic states, as observed here. This highlights the added value of combining BMD CI analysis with ToxPi visualization for more comprehensive insight. We recognize that this study is not completely comprehensive and does not represent the full complexity of multiplexed assays. However, the findings provide a proof of concept for applying these tools to datasets where binary outputs alone may overlook subtle patterns and interactions among endpoints. If this approach was applied to older assay data, clustering patterns similar to those observed here may emerge. However, the integration of metabolic state‐specific responses and the flexibility of endpoint weighting offered by ToxPi significantly enhance the interpretive power, particularly for comparative potency assessments.

## 5 Conclusion

In summary, multiplex assays are incorporating new analysis systems and machine learning algorithms to enhance biological insights and optimize data visualization for maximum information extraction. This methodology adeptly refines compound analysis by leveraging BMD CI to provide robust potency assessments, utilizing ToxPi weightings to capture underlying MoA, and incorporating cluster analysis alongside toxicity ranking to enable nuanced and detailed cross‐comparisons between diverse multiplexed datasets. Although further research is necessary to establish a standardized system for applying these methods across multiple assays, the methodology presented here demonstrates a clear and robust ability to compare the analysis of compounds and their potencies across multiple endpoints, with or without metabolic activation. Additionally, the weighting percentages for each biomarker remain the same for these analyses. Ongoing multiplex assay analyses using GeneToxPi with BMD on genotoxicity data aim to address these issues by developing a comprehensive weighting system for multiple endpoints across diverse genotoxic assays.

To establish these weightings, each individual genotoxic assay would require an increase in the number of reference compounds with known modes of action being used per analysis, and more biomarkers would require curation/processing to develop and apply a weight‐of‐evidence approach to weighing biomarkers. Health Canada performed this across multiple assays in one assessment; however, this focused on weightings of entire assays as opposed to individual biomarkers (Fortin et al. 2023). As alluded to earlier, more data from an extensive collective effort will help develop a biological and regulatory rationale for the weightings applied, especially when considering whether to prioritize in vivo vs. in vitro, atypical vs. early repairable indicator‐type responses, immediate vs. 4‐h vs. 24‐h responses, etc. Interpreting BMD CIs is a crucial aspect of modeling dose–response data across various assays.

The integration of BMD analysis with ToxPi visualization represents a pivotal advancement in the refinement of assay evaluation and compound hazard assessment. Each assay implemented requires a well‐designed protocol subjected to rigorous evaluation from statistical, mathematical, biological, and genetic perspectives while aligning with regulatory frameworks. These efforts enable the development of optimized analytical pipelines that leverage ToxPi's visualization capabilities to advance comparative techniques for numerous compounds in future research. Integrating BMD analysis with ToxPi enhances data interpretation, minimizes information loss, and addresses the limitations of current compound hazard assessment methodologies, paving the way for more robust and predictive approaches.

## Conflicts of Interest

All original benchtop experiments were performed at Litron Laboratories, which is a pharmaceutical firm that has patents covering the MultiFlow flow‐cytometric‐based assay assessed in this experiment and is commercially sold to customers as the MultiFlow DNA Damage Kit—p53, γH2AX, Phospho‐Histone H3.

## Supporting information


**Figure S1.** (A–H) – A collection of BMDs 90% CIs generated with a CES of 0.5 using PROAST v65.5 across TK6 cells exposed to 10 genotoxicants analyzing biomarkers: (A) 4 h γH2aX S9−, (B) 4 h P53 S9−, (C) 24 h γH2aX S9−, (D) 24 h p53 S9−, (E) 4 h γH2aX S9+, (F) 4 h P53 S9+, (G) 24 h γH2aX S9+, and (H) 24 h p53 S9+ response in the MultiFlow assay where higher log_10_(BMD) values indicate compounds with lower response.
