# Supplementary material for: Synthesizing Genotoxicity Results in the MultiFlow Assay With Point‐of‐Departure Analysis and ToxPi Visualization Techniques
Source: Environ Mol Mutagen. 2025 Mar 13;66(3):122–33. doi: 10.1002/em.70003 (PMC11986802; doi:10.1002/em.70003)
Supplement: Supplementary file 1 — Figure S1. (A–H) – A collection of BMDs 90% CIs generated with a CES of 0.5 using PROAST v65.5 across TK6 cells exposed to 10 genotoxicants analyzing biomarkers: (A) 4 h γH2aX S9−, (B) 4 h P53 S9−, (C) 24 h γH2aX S9−, (D) 24 h p53 S9−, (E) 4 h γH2aX S9+, (F) 4 h P53 S9+, (G) 24 h γH2aX S9+, and (H) 24 h p53 S9+ response in the MultiFlow assay where higher log10(BMD) values indicate compounds with lower response. [file EM-66-122-s001.zip › Supplementary Figure A.docx]

**Supplementary Figure A**


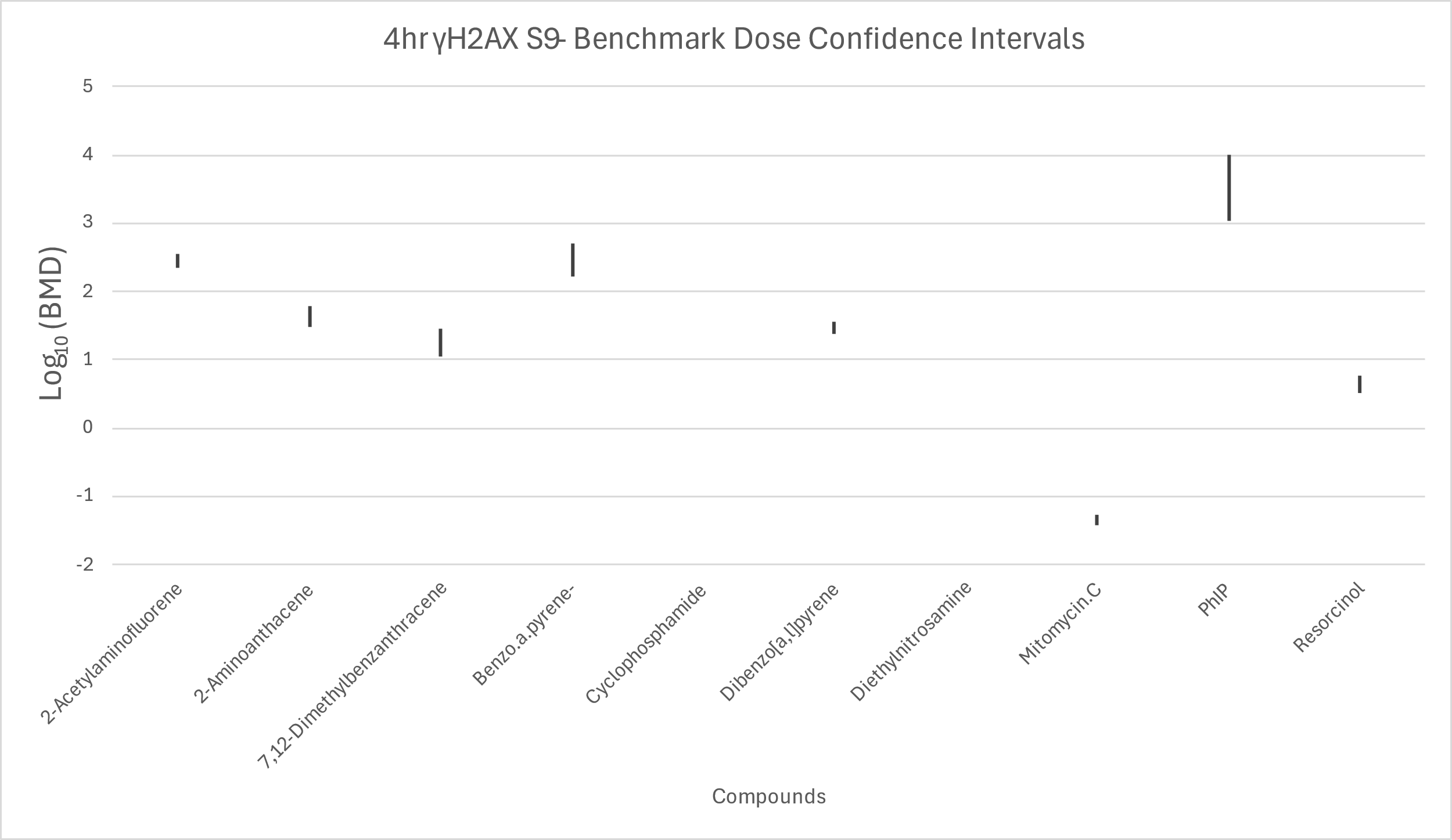


**Supplementary Figure B**

**Supplementary Figure**
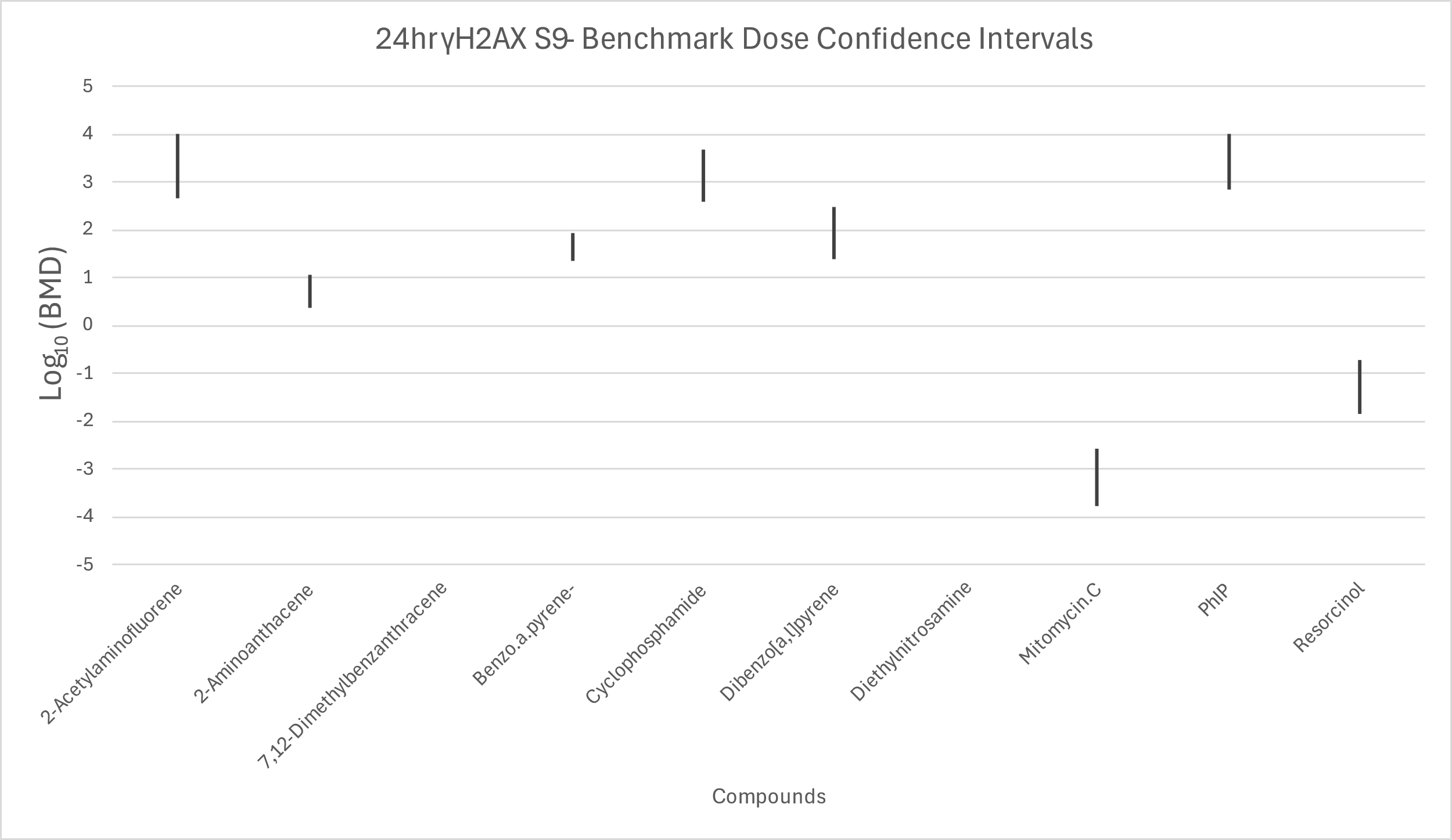
**C**

**Supplementary Figure** **D**

**Supplementary Figure E**


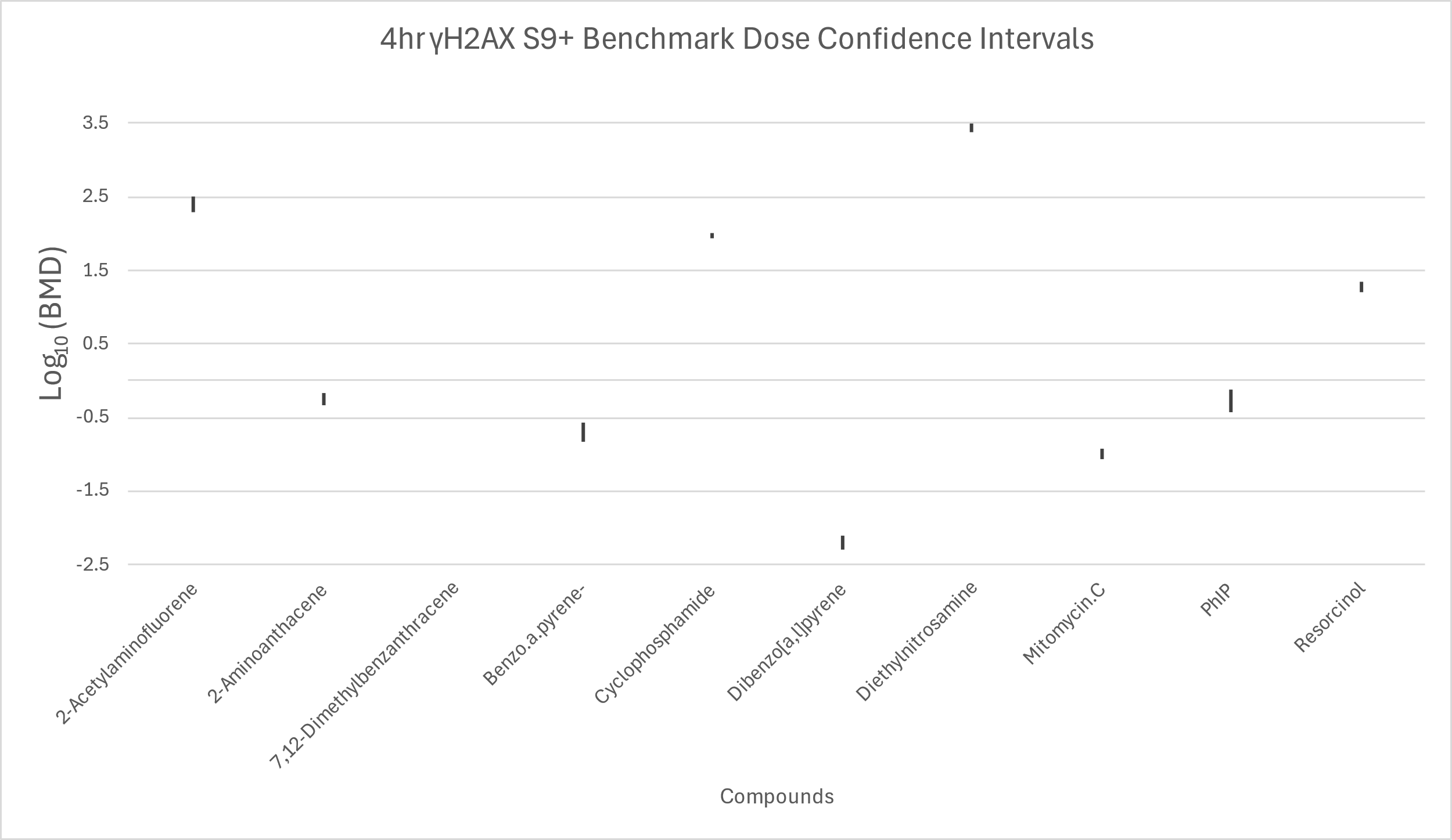


**Supplementary Figure F**

**Supplementary Figure G**


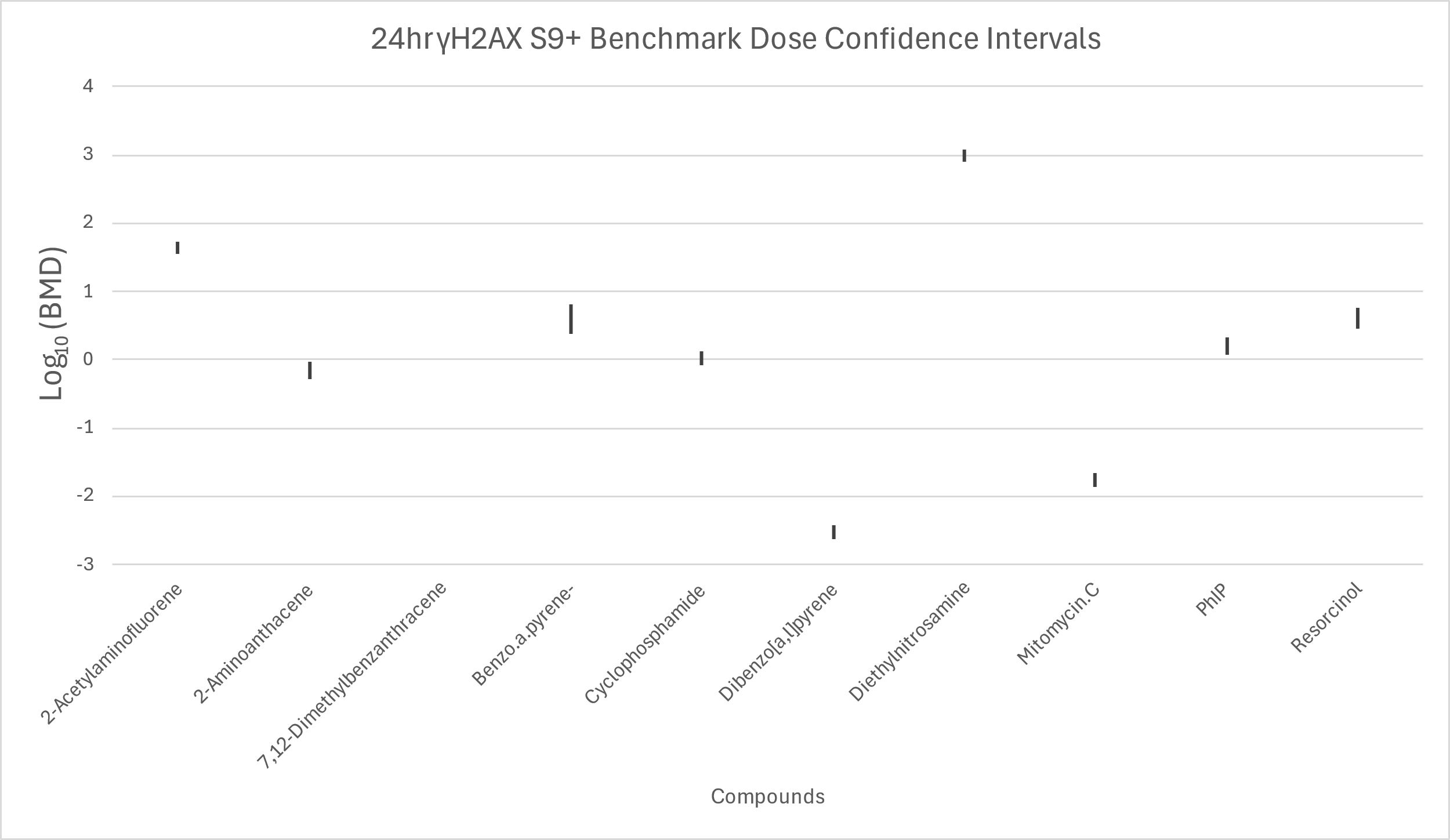


**Supplementary Figure H**

**Supplementary figure 1 (A – H)** – A collection of BMDs 90% CIs generated with a CES of 0.5 using PROAST v65.5 across TK6 cells exposed to 10 genotoxicants analyzing biomarkers: (A) 4hr γH2aX S9-, (B) 4hr P53 S9-, (C) 24hr γH2aX S9-, (D) 24hr p53 S9-, (E) 4hr γH2aX S9+, (F) 4hr P53 S9+, (G) 24hr γH2aX S9+, (H) 24hr p53 S9+ response in the MultiFlow® assay where higher log_10_(BMD) values indicate compounds with lower response.
