# Supplementary figures and images for: Synthesizing Genotoxicity Results in the MultiFlow Assay With Point‐of‐Departure Analysis and ToxPi Visualization Techniques
Source: Environ Mol Mutagen. 2025 Mar 13;66(3):122–33. doi: 10.1002/em.70003 (PMC11986802; doi:10.1002/em.70003)

### 24hr $\gamma$ H2AX S9+ Benchmark Dose Confidence Intervals

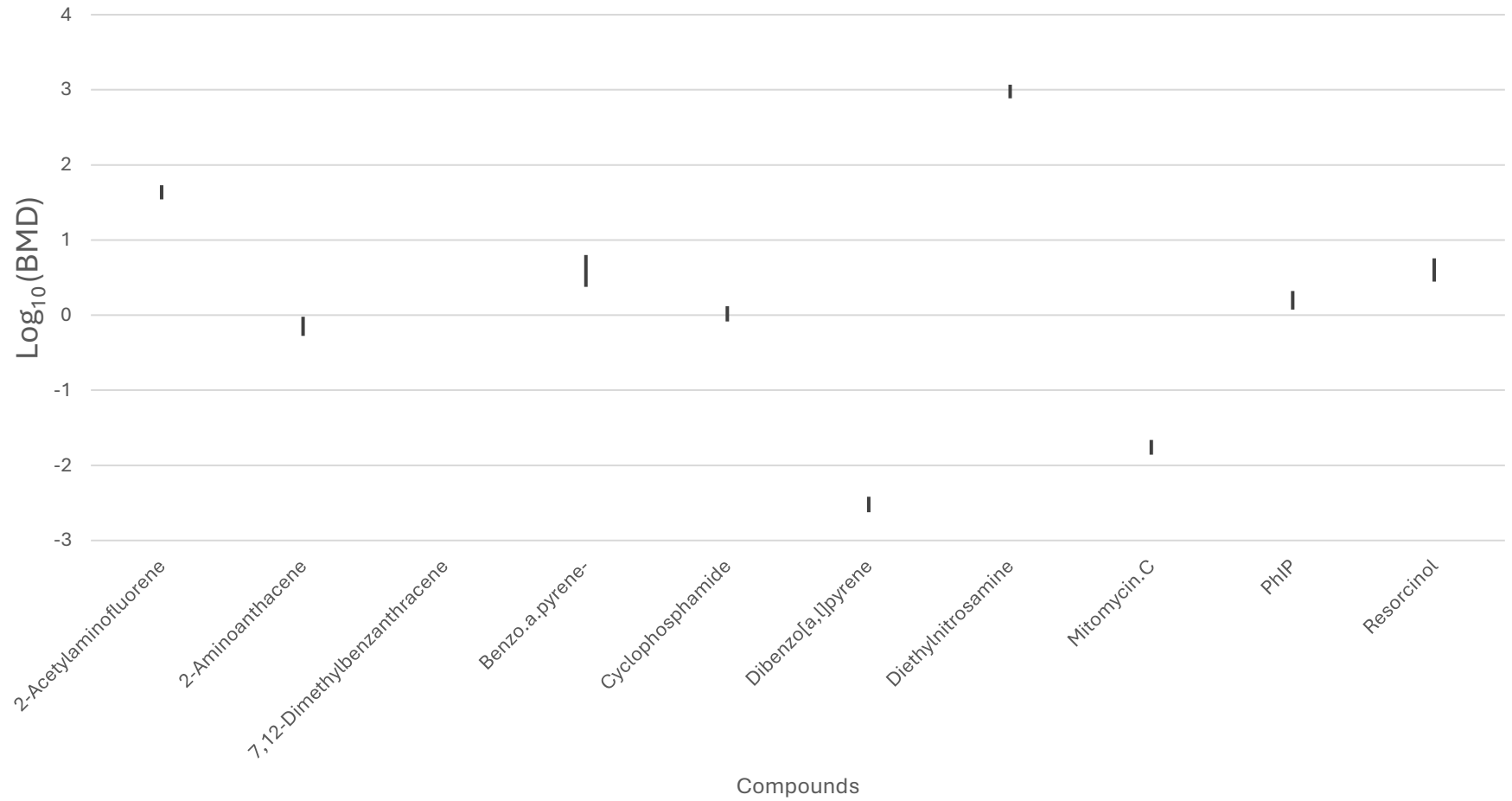

Supplement: Supplementary file 1 — Figure S1. (A–H) – A collection of BMDs 90% CIs generated with a CES of 0.5 using PROAST v65.5 across TK6 cells exposed to 10 genotoxicants analyzing biomarkers: (A) 4 h γH2aX S9−, (B) 4 h P53 S9−, (C) 24 h γH2aX S9−, (D) 24 h p53 S9−, (E) 4 h γH2aX S9+, (F) 4 h P53 S9+, (G) 24 h γH2aX S9+, and (H) 24 h p53 S9+ response in the MultiFlow assay where higher log10(BMD) values indicate compounds with lower response. [file EM-66-122-s001.zip › Sup G -24hr γH2AX S9+.pdf]

24hr p53 S9+ Benchmark Dose Confidence Intervals

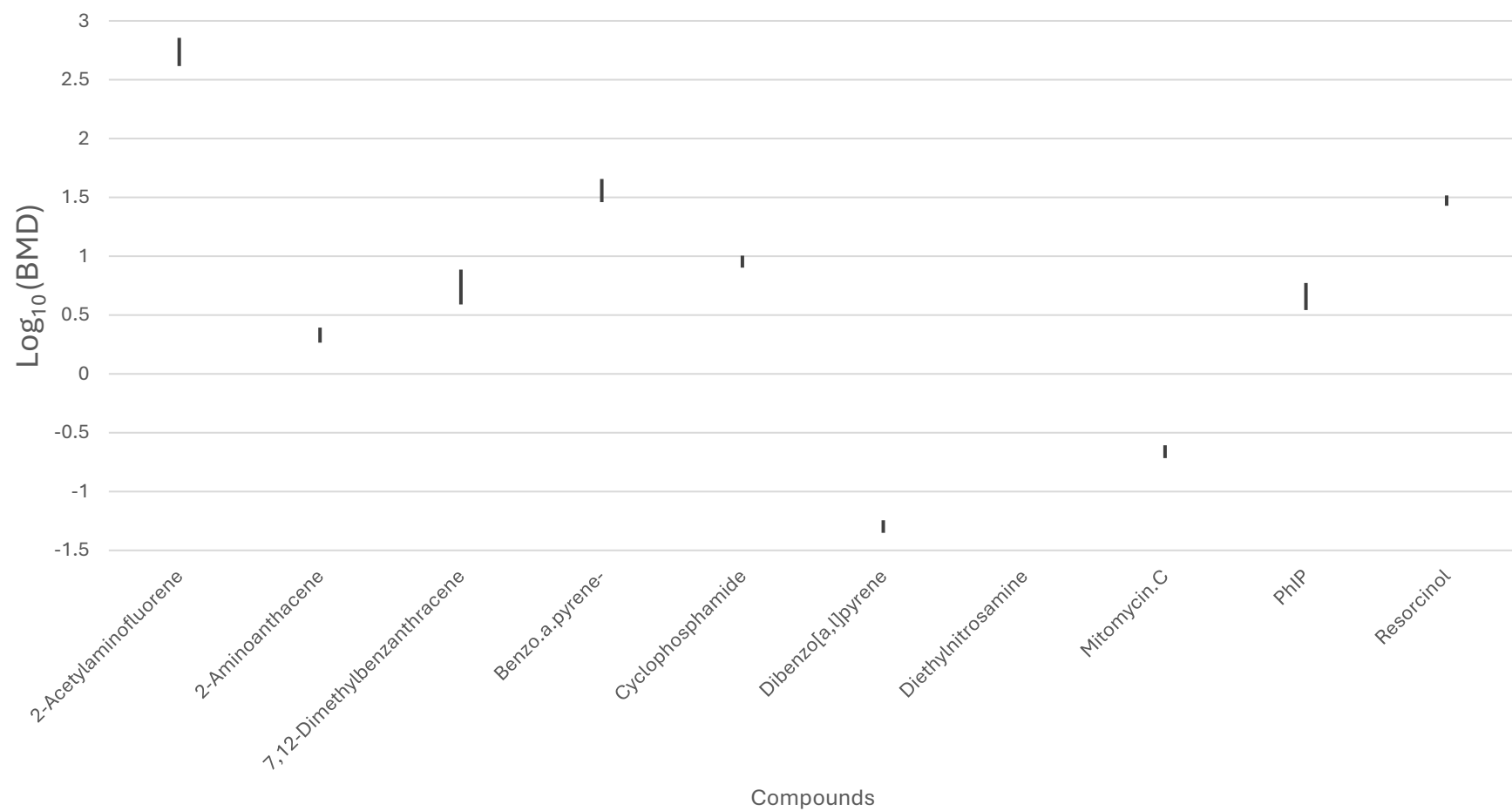

Supplement: Supplementary file 1 — Figure S1. (A–H) – A collection of BMDs 90% CIs generated with a CES of 0.5 using PROAST v65.5 across TK6 cells exposed to 10 genotoxicants analyzing biomarkers: (A) 4 h γH2aX S9−, (B) 4 h P53 S9−, (C) 24 h γH2aX S9−, (D) 24 h p53 S9−, (E) 4 h γH2aX S9+, (F) 4 h P53 S9+, (G) 24 h γH2aX S9+, and (H) 24 h p53 S9+ response in the MultiFlow assay where higher log10(BMD) values indicate compounds with lower response. [file EM-66-122-s001.zip › Sup H -24hr p53 S9+.pdf]

### 4hr $\gamma$ H2AX S9- Benchmark Dose Confidence Intervals

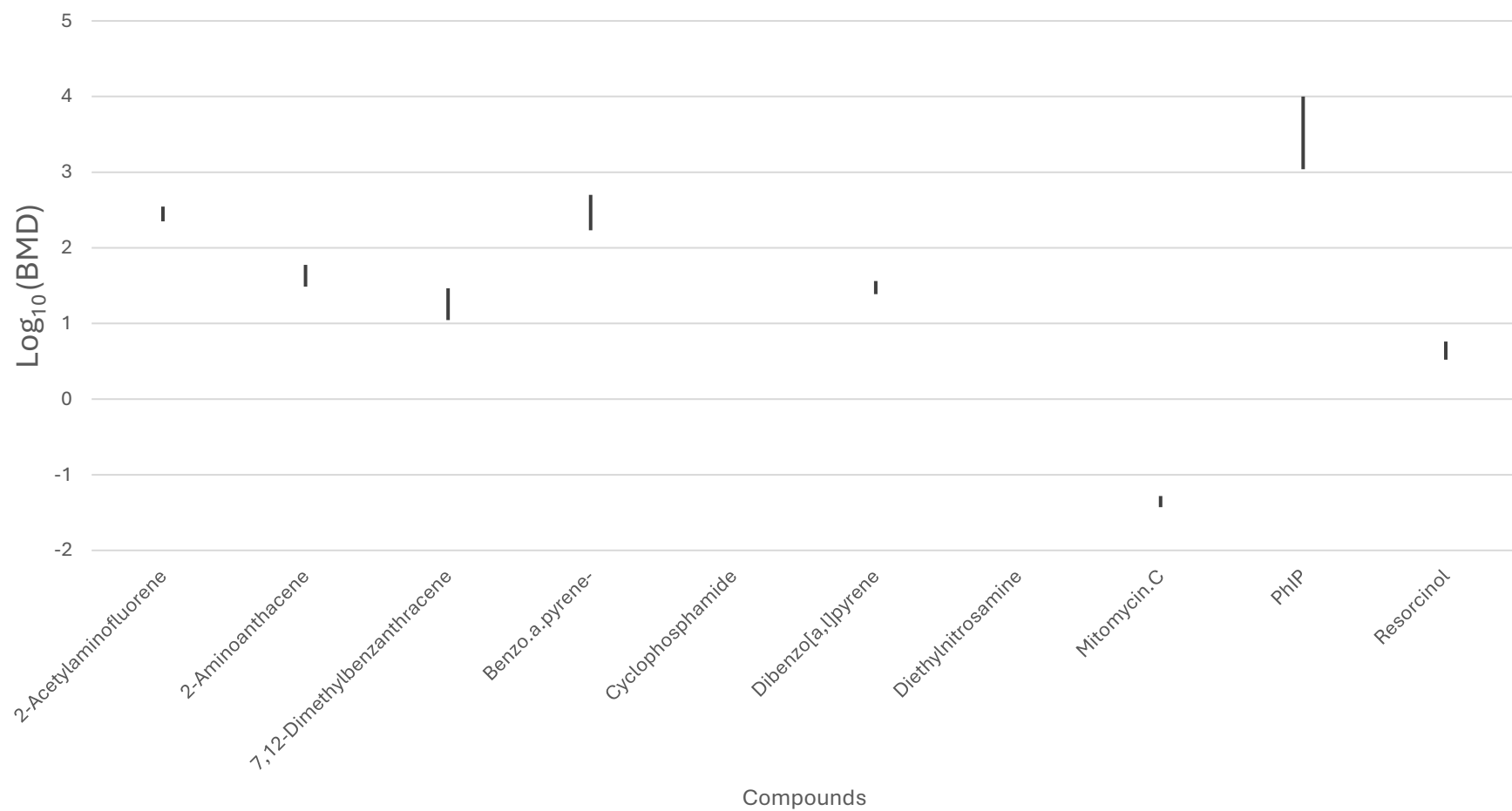

Supplement: Supplementary file 1 — Figure S1. (A–H) – A collection of BMDs 90% CIs generated with a CES of 0.5 using PROAST v65.5 across TK6 cells exposed to 10 genotoxicants analyzing biomarkers: (A) 4 h γH2aX S9−, (B) 4 h P53 S9−, (C) 24 h γH2aX S9−, (D) 24 h p53 S9−, (E) 4 h γH2aX S9+, (F) 4 h P53 S9+, (G) 24 h γH2aX S9+, and (H) 24 h p53 S9+ response in the MultiFlow assay where higher log10(BMD) values indicate compounds with lower response. [file EM-66-122-s001.zip › Sup A - 4hr γH2AX S9-.pdf]

4hr p53 S9- Benchmark Dose Confidence Intervals

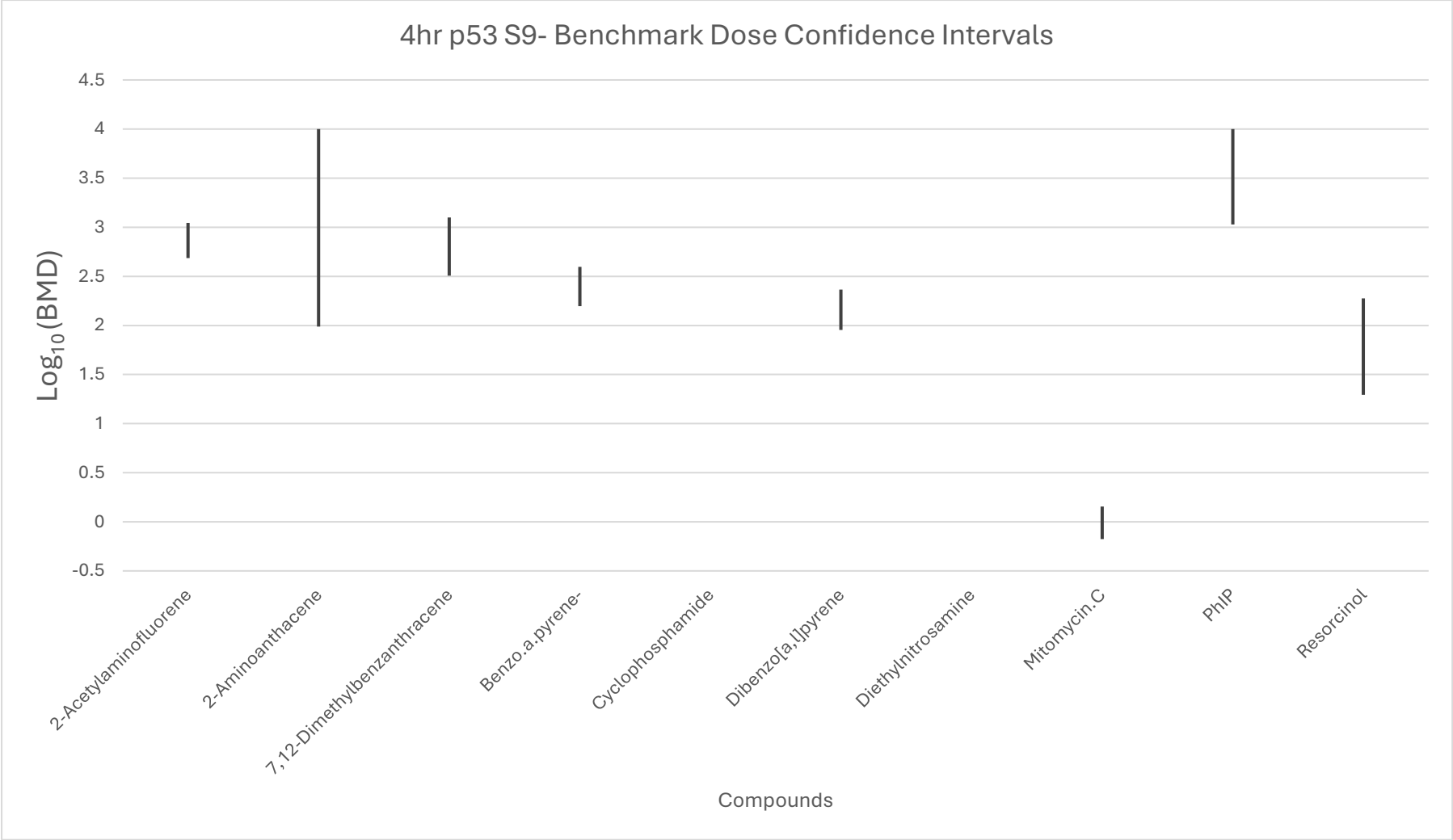

Supplement: Supplementary file 1 — Figure S1. (A–H) – A collection of BMDs 90% CIs generated with a CES of 0.5 using PROAST v65.5 across TK6 cells exposed to 10 genotoxicants analyzing biomarkers: (A) 4 h γH2aX S9−, (B) 4 h P53 S9−, (C) 24 h γH2aX S9−, (D) 24 h p53 S9−, (E) 4 h γH2aX S9+, (F) 4 h P53 S9+, (G) 24 h γH2aX S9+, and (H) 24 h p53 S9+ response in the MultiFlow assay where higher log10(BMD) values indicate compounds with lower response. [file EM-66-122-s001.zip › Sup B - 4hr p53 S9-.pdf]

24hr  $\gamma$ H2AX S9- Benchmark Dose Confidence Intervals

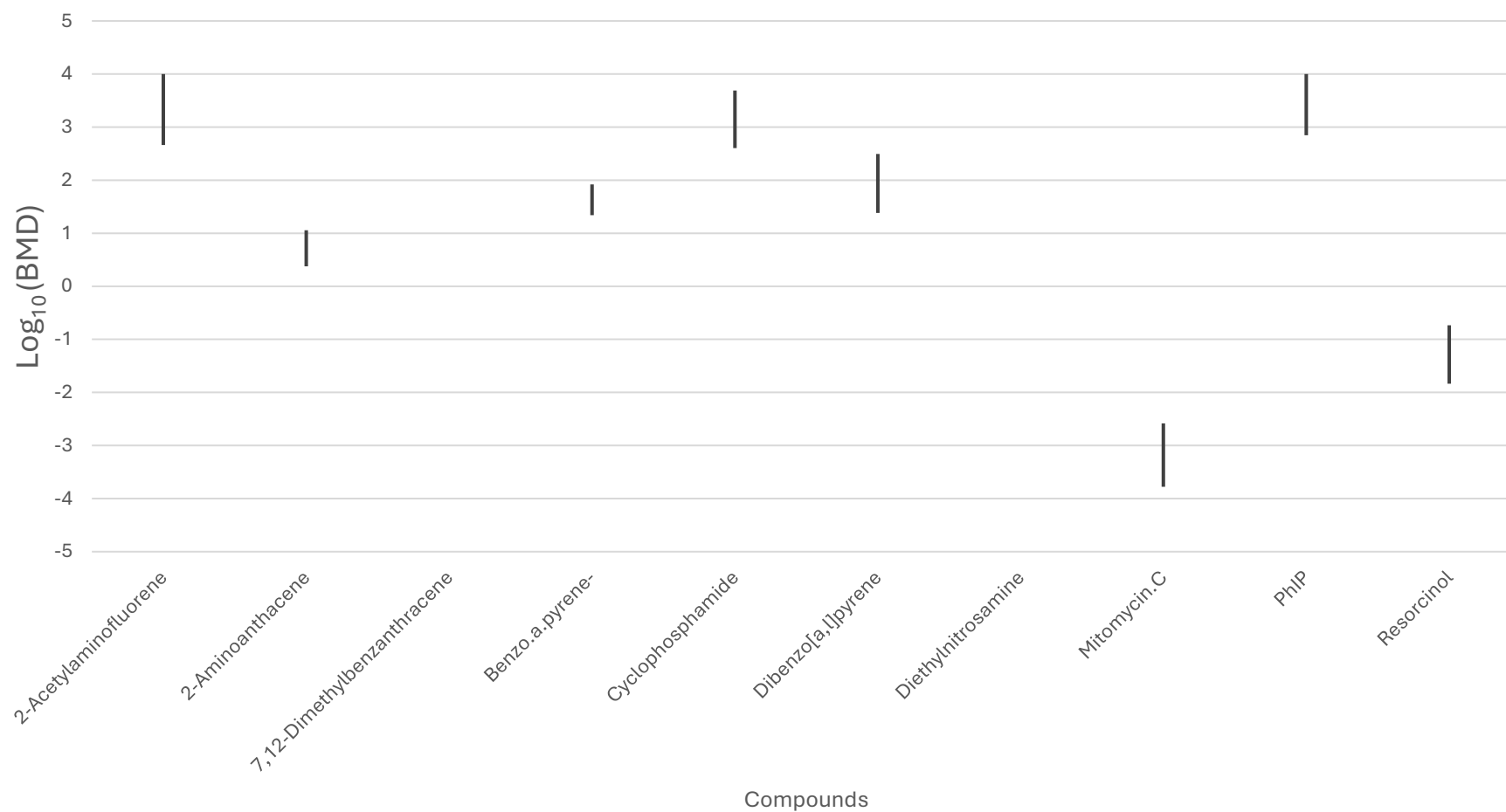

Supplement: Supplementary file 1 — Figure S1. (A–H) – A collection of BMDs 90% CIs generated with a CES of 0.5 using PROAST v65.5 across TK6 cells exposed to 10 genotoxicants analyzing biomarkers: (A) 4 h γH2aX S9−, (B) 4 h P53 S9−, (C) 24 h γH2aX S9−, (D) 24 h p53 S9−, (E) 4 h γH2aX S9+, (F) 4 h P53 S9+, (G) 24 h γH2aX S9+, and (H) 24 h p53 S9+ response in the MultiFlow assay where higher log10(BMD) values indicate compounds with lower response. [file EM-66-122-s001.zip › Sup C -24hr γH2AX S9-.pdf]

24hr p53 S9- Benchmark Dose Confidence Intervals

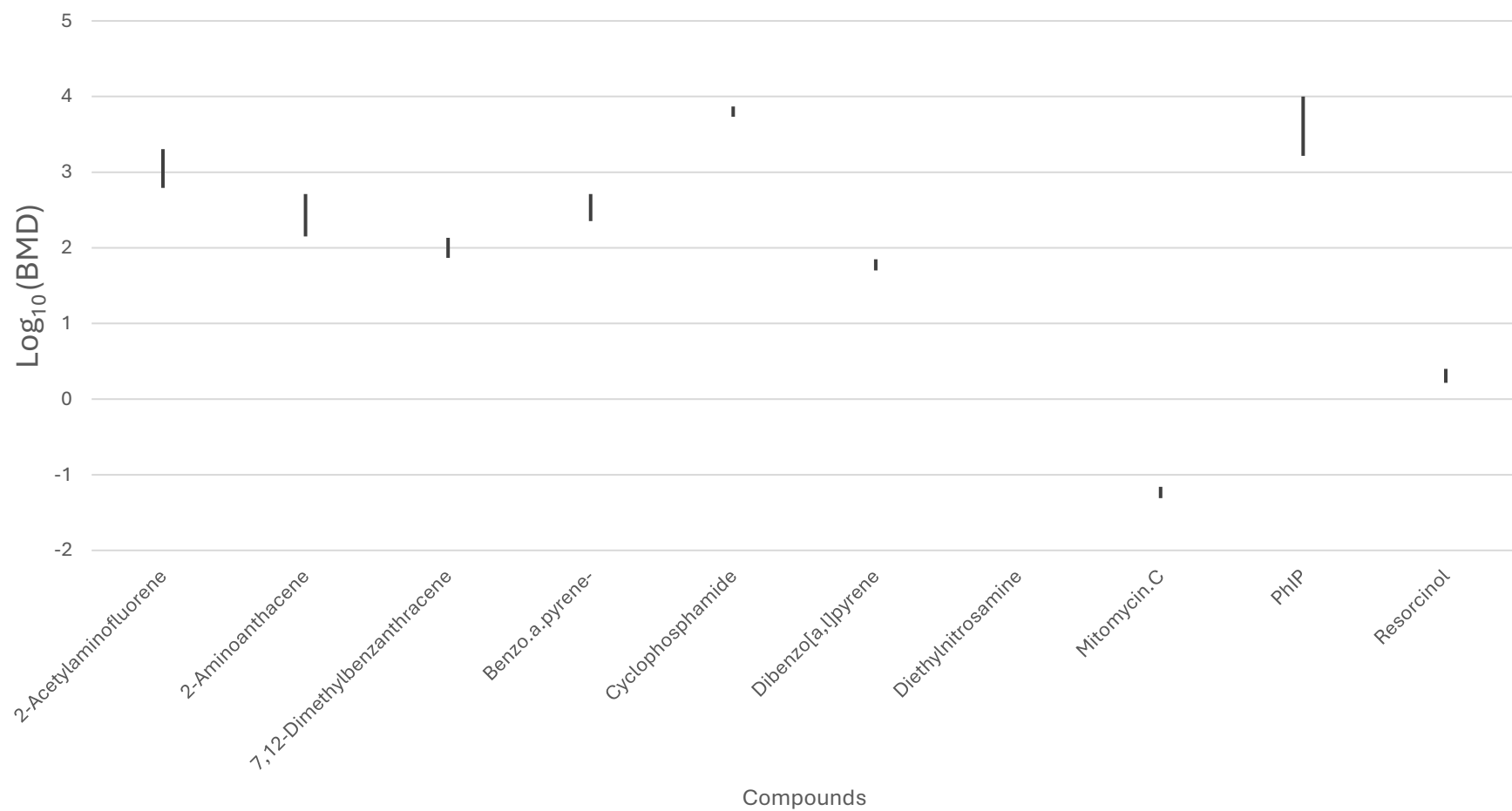

Supplement: Supplementary file 1 — Figure S1. (A–H) – A collection of BMDs 90% CIs generated with a CES of 0.5 using PROAST v65.5 across TK6 cells exposed to 10 genotoxicants analyzing biomarkers: (A) 4 h γH2aX S9−, (B) 4 h P53 S9−, (C) 24 h γH2aX S9−, (D) 24 h p53 S9−, (E) 4 h γH2aX S9+, (F) 4 h P53 S9+, (G) 24 h γH2aX S9+, and (H) 24 h p53 S9+ response in the MultiFlow assay where higher log10(BMD) values indicate compounds with lower response. [file EM-66-122-s001.zip › Sup D -24hr p53 S9-.pdf]

### 4hr $\gamma$ H2AX S9+ Benchmark Dose Confidence Intervals

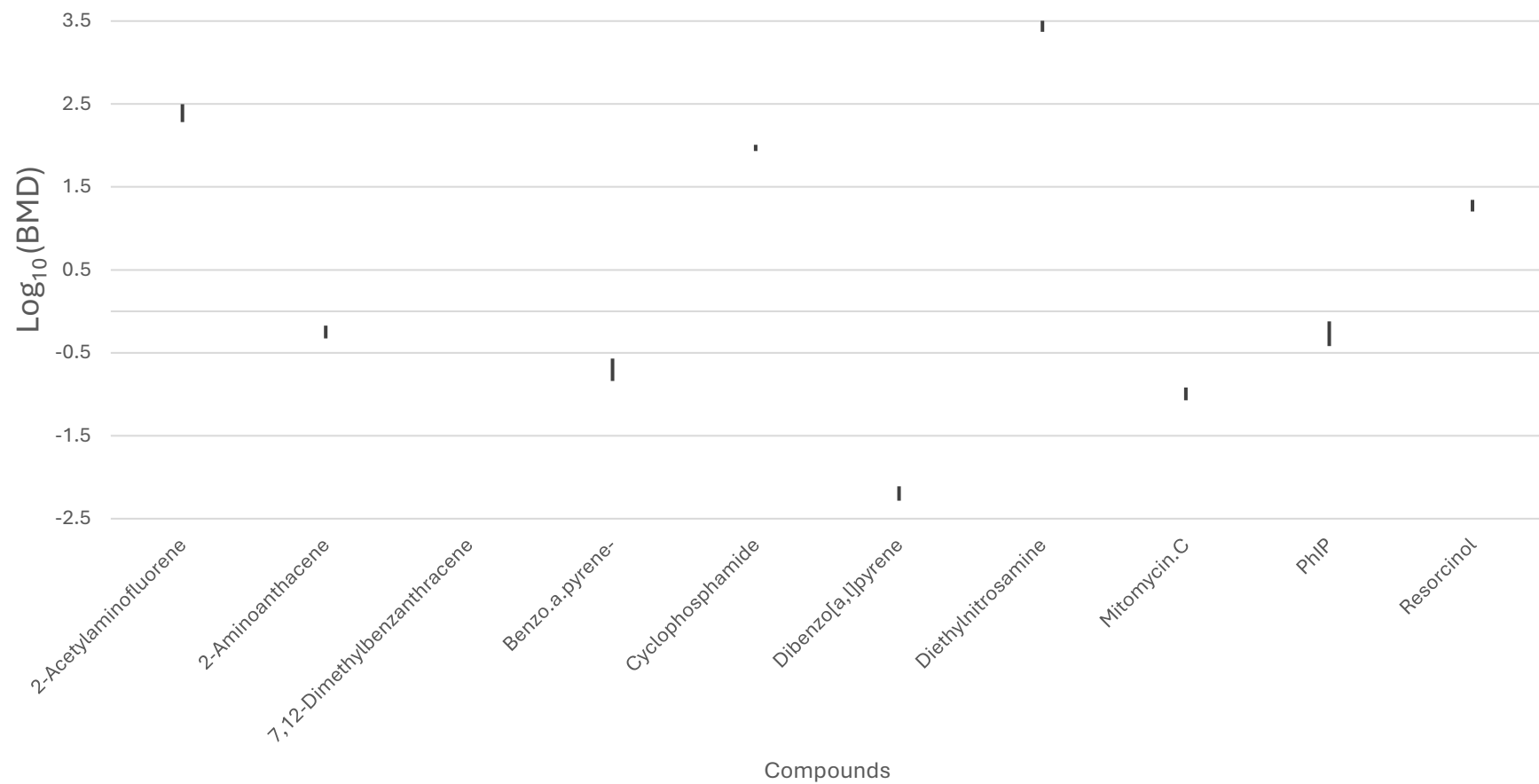

Supplement: Supplementary file 1 — Figure S1. (A–H) – A collection of BMDs 90% CIs generated with a CES of 0.5 using PROAST v65.5 across TK6 cells exposed to 10 genotoxicants analyzing biomarkers: (A) 4 h γH2aX S9−, (B) 4 h P53 S9−, (C) 24 h γH2aX S9−, (D) 24 h p53 S9−, (E) 4 h γH2aX S9+, (F) 4 h P53 S9+, (G) 24 h γH2aX S9+, and (H) 24 h p53 S9+ response in the MultiFlow assay where higher log10(BMD) values indicate compounds with lower response. [file EM-66-122-s001.zip › Sup E -4hr γH2AX S9+.pdf]

4hr p53 S9+ Benchmark Dose Confidence Intervals

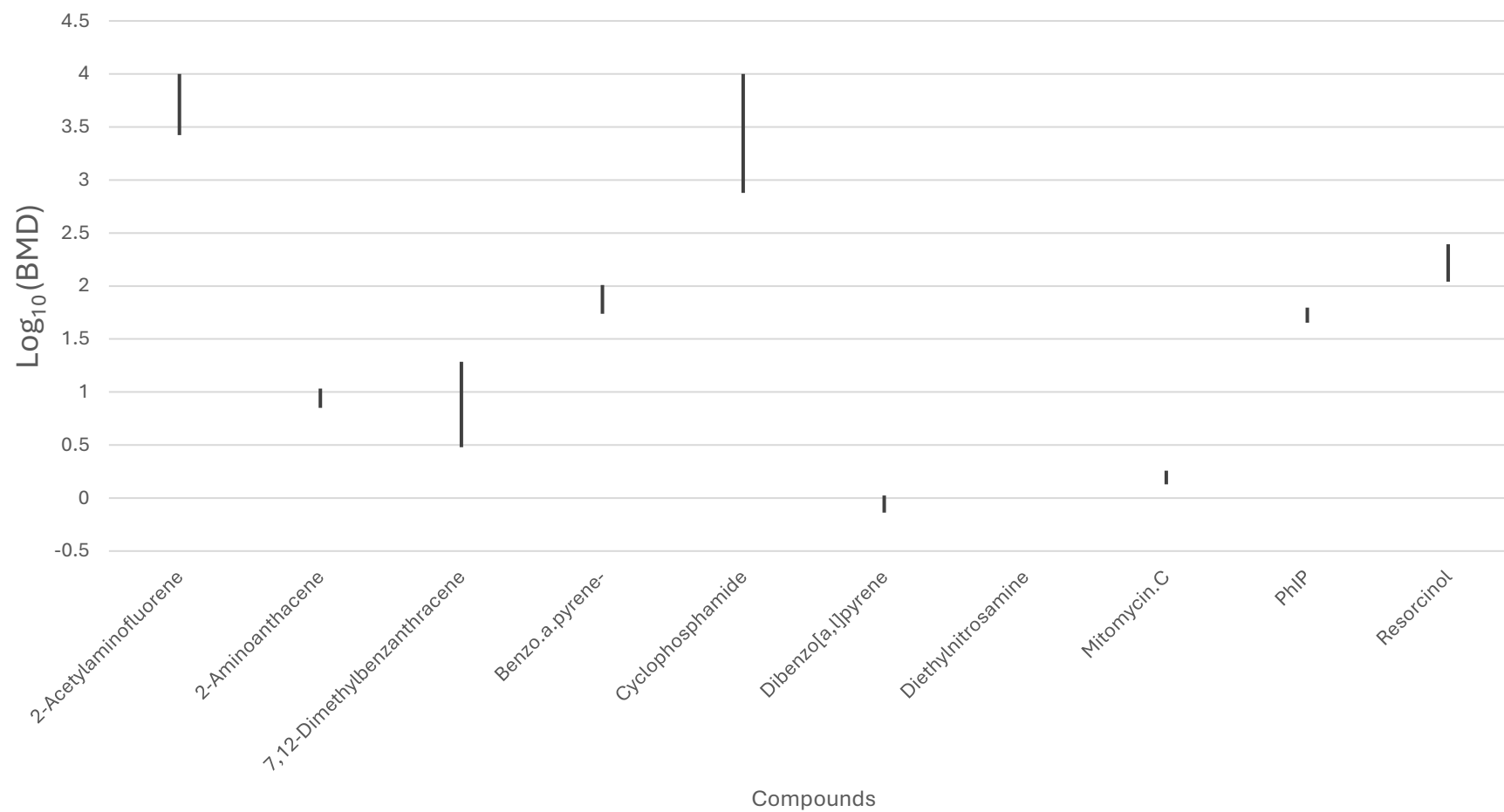

Supplement: Supplementary file 1 — Figure S1. (A–H) – A collection of BMDs 90% CIs generated with a CES of 0.5 using PROAST v65.5 across TK6 cells exposed to 10 genotoxicants analyzing biomarkers: (A) 4 h γH2aX S9−, (B) 4 h P53 S9−, (C) 24 h γH2aX S9−, (D) 24 h p53 S9−, (E) 4 h γH2aX S9+, (F) 4 h P53 S9+, (G) 24 h γH2aX S9+, and (H) 24 h p53 S9+ response in the MultiFlow assay where higher log10(BMD) values indicate compounds with lower response. [file EM-66-122-s001.zip › Sup F -4hr p53 S9+.pdf]
